# Cases and Remarks on Hydrocephalus Internus, with a Description of a Morbid Appearance of the Cerebrum

**Published:** 1802-08-01

**Authors:** J. B. Davis

**Affiliations:** Member of the Royal College of Surgeons, &c.


*Cases and Remarks on Hydrocephalus Internus, with a Description of a morbid Appearance of the Cerebrum; communicated by* Mr. J. B. Davis, *Member of the Royal College of Surgeons, &c.*

April 4th, 1802. Anne Smith, five years of age, was feized with convulfive motions over her whole body. The hiftory of her illnefs was this. She had been during two months gradually drooping; feverifh heats came on towards night; the tongue was white and dry, an univerfal languor prevailed, vifion became imperfect, and the appetite diminifhed. About a fortnight before I faw her, fhe began to complain of head-ach, which was foon fucceeded by a flight fit and a temporary exacerbation of the other fymptoms. Prior to the firft approach of this complaint the child enjoyed perfect health, and her mother informed me, that fhe never had had the head-ach till five or fix weeks after the commencement of the prefent indifpofition. I examined the head and found it very large, having the fontanelles widely extended; but from the apparent preffure on the brain, and the agitation induced by convulfive efforts, I could not pofitively decide what the cafe was, though I ftrongly fufpected, from the narrative of Mrs. Smith, that it muft be Hydrocephalus Internus. At the next vifit, however, I was able to fatisfy myfelf of the truth of my conjecture the preceding evening; and the following train of fymptoms, which I then afcertained without any difficulty, will, I believe, be confidered as ample proofs of the exiftence of that difeafe. Dilated pupils, flow and oppreffed pulfe, thirft, lofs of appetite, coftivenefs, pain in the head, great drowfinefs, but difturbed fleep, white and dry tongue, coldnefs of the feet, dimnefs of fight, &c. Three or four fits came on in all; and as the veffels of the encephalon became diftended by a determination of blood to the head, the dimnefs of fight and dilatation of the pupils increafed. The complaint infidioufly gained ground from the fecond convulfion; and morbid action, which had hitherto been flow and progreffive, doubtlefs now acquired activity, and filled the cavities of the brain with a preternatural quantity of fluid. The pulfation at the temporal arteries was very ftrong, though flow; and if I might judge from that, and the pain which I produced by raifing or moving the head to either fide, the meninges of the encephalon, as well as the brain itfelf, were in a ftate of high inflammation. My little patient would fometimes give rational anfwers, but oftener be altogether infenfible to what was faid to her. The ftupor which fhe generally had, a good deal refembled the delirium that attacks perfons with inflammation tion of the pia mater from injuries of the head. I regretted that nothing had been hitherto done for the child's relief, and was determined, without delay, to apply the moſt powerful remedies.

I opened the temporal artery the firſt evening I was conſulted, and took away four ounces of blood; ordered a bliſter to be put to the top of the head, and half a drachm of mercurial ointment to be rubbed into the nape of the neck immediately. As the bowels were in a confined ſtate, and had been ſo ſome time, I preſcribed three grains of calomel and a ſolution of magnes. vit. to produce ſeveral briſk evacuations. The next morning, April 5, I again opened the temporal artery, and obtained four or five more ounces of blood. The bliſter had acted extremely well; and being anxious to irritate the ſurface and keep up a diſcharge, I removed the cuticle and dreſſed the expoſed part with ungt. ſabinæ. Ulceration of the ſcalp is generally ſerviceable in organic affections of the cerebrum; and as a quantity of blood was previouſly drawn from the veſſels of the head, I had an idea that abſorption would take place more readily, and I accordingly promoted counter irritation by gentle ſtimuli. Local fullneſs being thus carried off, there could be no danger of keeping up inflammation, by determining to a ſurface ſo nearly connected with the encephalon. Antimonials adminiſtered to excite nauſea ſometimes do good, and I tried them with benefit in this caſe. I directed a ſolution of the antimon. tart. to be made and given at proper intervals till the ſtomach either became uneaſy or rejected it. It was thus prepared: ℞. Antimon. tart. gr. iij. Aq. pur. ℥xv. ſpt. cinnam. ℥j. M. capt. cochlear parv. 3tis horis. Ointment repeated. On the evening of the ſecond day pulſe 96; ſlept better, and did not appear to be in ſuch pain. April 6th, Was rather ſick; pulſe 90; eyes dilated as before; ſlept tolerably well during the night, and awoke with more compoſure; coldneſs of the extremities gone off; ointment repeated; antimon. ſolution given once in ſix hours. April 7th, Nauſea, but no vomiting; could ſuffer the head to be elevated and moved from ſide to ſide; pulſe 86; ſlept very well; was rational; coſtiveneſs ſupervened, for which I preſcribed a mercurial purge, conſiſting of two grains of calomel and ſix of jalap; dimneſs of ſight remained; ointment repeated. April 8th, Opening medicine had not operated; head uneaſy; pulſe 94; very thirſty; hot ſkin; diſturbed ſleep. My patient was evidently worſe, and required ſome immediate evacuations. I preſcribed an aperient enema, compoſed of ſal cathart. ol. Ricin. et dec. com. pro enemate, and directed three or four leeches to be applied behind each ear. In the evening I perceived an amendment, the pulſe being reduced to 86, and the head eafier. Advifed two fcruples of the ointment to be rubbed into the neck; and in place of the antimonial folution, a grain of calomel at bed-time. A drachm of aq. ammon. acet. was given once in fix hours, in order to allay feverifh fymptoms. April 9th, Head eafy; pulfe 75; lefs heat; flept very well. The effect of the mercury being flow, I ordered the child to be put into warm falt water, with a view of expediting its influence on the gums. Calomel repeated. An enema was adminiftered, viz. ℞. Dec. com. pro enemat. ℔fs. ol. Ricini ℥fs. ft. enema. April 10th, Pulfe 70; flept extremely well; gentle perfpirations; head not at all painful; talked rationally; complained now and then of being very languid. I prefcribed, ℞. Mift. camph. ℥iijfs. fpt. æther vit. comp. ʒj. fyr. tolut. ʒij. M. cochlear I amp. quando urgeant languores. Ointment repeated. April 11th, Pulfe 70; flept well; head eafy; talked fenfibly; took freely of beef tea; fkin foft and moift; gentle perfpirations during the night; mouth fore. Directed the blifter to be ftill kept open, and the fame plan continued. April 12th, Slight ptyalifm; head perfectly free from pain; had good nights; pulfe 72; pupils more irritable; fight imperfect as before; fat up two hours, and converfed rationally, but could not remember any thing that was faid the preceding day. Plan continued. April 13th, Pulfe 70; fight improved; pupils more contractile; flept well; head light, but free from pain; ptyalifm increafing; bowels rather confined. Ordered the aperient enema to be adminiftered, and the ointment left off. April 14th, Appetite returning; ptyalifm moderate; pulfe 70; fight better; head eafy; took beef tea and calve's foot jelly feveral times in the courfe of the day. April 15th, Pupils dilated; head very light; pulfe languid; fpitting moderate. Prefcribed ℞. Conf. aromat. ʒij. aq. cinnam. ʒj. fpt. ammon. co. ʒj. aq. pur. ℥iv. fyr. fimp. ʒij. M. cochlear I amp. fæpe fumend. April 16th, Pulfe languid; head more comfortable; fight improving; ptyalifm abating. Medicine repeated. April 17th, Much the fame; directed the blifter to be dreffed with healing ointment. April 18th, Very fenfible; pulfe ftill low; head eafy; took much nutriment, which confifted of jelly, beef tea, light puddings, &c. April 19th and 20th, Much the fame. April 21ft, Pulfe acquiring ftrength; memory indifferent; head very eafy; fat up three or four hours; fight much better; Pupils more contractile; blifter almoft healed. April 23d, Better in all refpects. April 26th, Gaining ftrength faft; good nights; bowels regular; eyes not fo dull. April 30th, Going on well. May 2d, Memory impaired; pupils contracted as in health; fight extremely good; walked about the room by herfelf. May 5th, Was fo much recovered recovered that I did not think it neceffary to fee her again. The only relict of the difeafe was failure of memory, which imperfection, I concluded, would be removed as the functions of the brain refumed their former power. She went into the country, at my requeft, and there remains. I am fince informed by her mother, that fhe acquires ftrength rapidly, but cannot recollect any thing that has occurred after the fpace of two or three days.

A little girl, about four years of age, became dejected, loft her appetite, and could not bear to be in company with other children, nor within the found of any noife. The mother did not much regard fuch fingular conduct until the child's health grew very indifferent, when fhe deemed it proper to have the opinion of a medical gentleman. I was the fecond perfon confulted upon this occafion, and foon difcovered that I had met with another cafe of Hydrocephalus Internus. On the 16th of April, 1802, the following fymptoms prevailed: Head-ach, oppreffed pulfe, white tongue, anorexia, conftant thirft, coftivenefs, dilated pupils, infenfibility; a great deal of pain fucceeded an attempt to turn the head to either fide, which generally inclined forwards. The little fufferer's hand alfo ftrongly pointed out the feat and extent of the difeafe, it being always placed upon the forehead or the top of the head.

Two months had elapfed fince the firft appearance of any thing extraordinary in the child's behaviour, which the mother at that period rather imputed to fingularity of temper than indifpofition. Her habit neverthelefs was fcrophulous. The eyes were prominent, head large, complexion delicate, upper lip thick, and the glands of the neck indurated. However trifling the inflammation of the encephalon or pia matter was at an early period of this complaint, I had no doubt of its having made great progrefs now. In fhort, every thing concurred to make me fuppofe that the cavities of the brain were completely diftended, and that confiderable preffure was thereby produced. The length of time which my patient had been ill, her weakly conftitution, and the protracted ftate of the complaint, all militated againft the profpect of recovery. I knew that it would be impoffible to fucceed unlefs I removed compreffion, and I dreaded left the evacuations neceffary for that purpofe fhould debilitate a fyftem already much exhaufted, and thus inftead of reftoring health render the cafe more dangerous. Yet the fuccefs I experienced in the former inftance from emptying the veffels of the head, encouraged me to adopt the fame plan again. I therefore began by opening the temporal artery, from which was obtained three ounces of blood; directed a blifter to be applied to the head, and half a drachm of ftrong mercurial ointment to be rubbed into the nape of the neck. An enema was alſo adminiſtered, viz. ℞. Decoct. com. ℔ß. mag. vitriol, prius decoct. ſolut. ℥ß. ol. Ricini ʒij. ft. Enema. On the 17th of April, I preſcribed the emetic ſolution, (ſee page 99), which in the courſe of a few hours had the effect of exciting nauſea; ordered the ointment to be repeated; and as the evacuations hitherto made were well ſuſtained, I took away three more ounces of blood from the temporal artery. In the evening the pulſe was very quick, but leſs oppreſſed; head ſtill uneaſy; ſkin hot; thirſt exceſſive; pupils dilated; diſturbed ſleep. On the 18th April, very coſtive; tongue white and dry; ſudden ſtarts; conſtant muttering; did not underſtand any thing which was ſpoken; pupils ſo inirritable that a candle put cloſe to the eyes did not occaſion contraction; pulſe weak, but rapid. I preſcribed a calomel bolus and a ſolution of mag. vit. a repetition of the ointment, and a warm ſalt bath. The bliſter during this time diſcharged freely. On the 19th, pulſe rapid; delirium; coldneſs of the extremities; partial perſpirations; breathing ſhort; frequent ſighs. The emetic mixture was diſcontinued, and this given in its place: ℞. Conf. aromat. ʒjß. ſpt. cinn. ℥j. miſt camph. ℥ivß. ſpt. ammon. co. gt. xx. ſyrupi ʒij. M. cochlear i amp. ſæpe ſumend. Ointment repeated. I directed a bliſter to be put on between the ſhoulders. The bowels were in a regular ſtate. On the 20th, humours of the eye appeared opaque; pupils dilated as before; pulſe 120; breathing ſhort and ſonorous; could not ſuffer the head to be moved; diſturbed ſleep. Medicines repeated. Unable to take any nutriment. On the 21ſt, pulſe ſo quick that the pulſations could not be numbered; feet and hands cold; ſubſultus tendinum; convulſions. The chance of doing good was now gone by, and every hope of recovery loſt; I therefore determined to afford that degree of palliation which appeared to be attainable in my patient's ſituation. On the 22d, convulſions returned very often; breathing became more difficult; pulſe intermitted; and in the afternoon ſhe expired.

### DISSECTION.

I took an opportunity of inſpecting the head after death, and carefully expoſed the meninges of the brain, in order to diſcover whether any morbid change had occurred in them, or if the encephalon *alone* was the ſeat of diſeaſe. The dura mater firmly adhered to the internal table of the ſkull, having its inner ſurface ſmooth, and every where detached from the pia mater, excepting at their venous communications. As ſoon as I reflected the firſt covering, I was delighted with the beautiful appearance of the pia mater. From a prodigious diſtenſion of the large veſſels, and a very general diſtribution of blood into minute minute ramifications, the whole fuperficies of the membrane which was then in view had affumed a florid afpect. Upon a farther examination, thofe duplicatures alfo, infinuating themfelves into the various convolutions of the cerebrum, partook of the fame colour, and altogether formed one of the fineft fpecimens of inflammation I ever recollect to have feen. There was no extravafation between the meninges to produce preffure, nor a want of the natural fecretion defigned for lubricating their furfaces and preventing adhefion; but feparating the hemifpheres of the brain with my fingers, to look at the corpus callofum, I perceived a fmall quantity of *a thick whitish fluid.* Of this I collected about half a tea fpoonful, and fearched along the proceffes of the pia mater for more, but without fuccefs. Having thus put afide the lateral parts of the cerebrum, and attentively obferved the middle portion of the medullary fubftance, I cut flowly through the fubftantia corticalis, in a convex direction, until I arrived at the vaulted arch made by the union of the medullary and cortical fubftances, where I ftopped to examine thofe portions of brain which were removed. The fubftantia alba had a reddifh hue, fomething like what would arife from a fubtle injection; and the fubftantia corticalis was darker than it is ufually found. Inftead of readily yielding to a flight ftroke of the knife, they both firmly refifted, and alfo poffeffed an unufual degree of tenacity. When I took a portion of either in my hand, the more accurately to inveftigate its ftructure, much force was required to detach the fmalleft piece; and I was aftonifhed to fee that the whole of what I laid afide was equally compact and fibrous. In continuing the diffection, I divided a vaft number of turgid veffels, and the nearer I approached the centrum ovale, the more perceptible was the rednefs. I expected to find a confiderable quantity of water in the ventricles; and in this I was not deceived, for the moment I punctured the lateral cavities, there iffued two table fpoons full of a difcoloured fluid, *not in the least coagulable by heat or acids.* The plexus choroides was drawn into folds, and had numerous little lumps in it of an exceeding hard nature. An experiment is commonly adverted to in a found ftate of the membrane to fhew thefe tubercles; but here morbid enlargement rendered them extremely confpicuous, and fo much fo, that there was no occafion to expand the plexus, or adopt any method to make the demonftration plainer. Each tubercle was certainly larger than the head of a pin*, and there were feveral clufters of them.

The

* Of a moderate fize.

The veins running on the fides of the lateral portions of the plexus, and uniting behind the glandula pinealis to difcharge themfelves by one trunk into the torcular herophili, were remarkably dilated. I believe the plexus choroides generally adheres to the pineal gland with confiderable firmnefs; and unlefs great care is taken, cannot be removed without laceration. The gland in this cafe was rather fwoln; and although I endeavoured for fome time to detach the plexus, my efforts proved unfuccefsful. On the delicate membrane which covers the corpora ftriata, thalami nervorum opticorum, infundibulum, fides of the fornix, &c. minute veffels were feen that are only difcernible by injections or difeafe. The mefenteric glands of this child were five or fix times larger than their natural fize, and contained a cafeous kind of fubftance.

I would now beg leave to afk, why are the optic nerves commonly relaxed in hydrocephalus internus? Does it depend upon an increafed quantity of fluid in the ventricles of the brain, or an interruption to the natural functions of that vifcus from any other caufe? Whether preffure be produced between the meninges of the encephalon, or in its cavities, not only the nerves of vifion, but the whole nervous fyftem, becomes affected. The fame phenomenon occurs if inflammation of the cerebrum, or even concuffion, takes place. Dilatation of the pupils is therefore, perhaps, firft attributable to morbid action in the brain, or its meninges, and fecondly to extravafation. It is probable, that at the beginning of hydrocephalus internus, a flight effufion happens *without previous inflammation* of the pia mater; but in all thofe cafes* which I have examined after death, that delicate membrane was invariably inflamed. The natural quantity of fluid thrown out into any cavity is extremely fmall; and in a healthy perfon, a mere adequacy to prevent agglutination, and facilitate the proper actions of the part. Serious confequences feldom, I conceive, follow fecretions moderately augmented; for if the conftitution be but tolerably vigorous, the furplus is foon removed by the activity of the abforbents.

In this complaint the tubercles of the plexus choroides are moftly enlarged, though I fufpect, like other fcrophulous affections, it has its origin in the lymphatics, which infenfibly becoming diftended, communicate inflammation to contiguous and fufceptible parts. The complexion of thofe children who are difpofed to hydrocephalus is ufually unhealthy, their conftitutions

* I have at different times infpected the heads of eighteen or twenty patients who have died of this difeafe, and always obferved the pia mater to be diftended with blood.

tutions weak, and circulation through the abforbent fyftem very imperfect. Perhaps it is inadmiffible to fay, that the brain I have given a defcription of was ftrumous, as I believe fuch a peculiar ftructure is rarely feen in dropfy of the head; though from the difeafed ftate of the mefenteric glands, and the ftrong characteriftic marks of fcrophula, I am much inclined to think it was a far advanced fpecimen of that peculiar inflammation.

Practitioners are unfortunately not confulted till the collection of water in the cavities of the brain is confiderable, and the pia mater prodigioufly loaded with blood. The general mode of treatment, therefore, however excellent it may be in principle, is frequently inefficacious, and in too many inftances incapable of affording the leaft relief. The profpect of recovery is at firft moft likely placed at a great diftance; and unlefs very potent remedies are immediately employed, death will fhortly put an end to the expectations of all. Should the lymphatic fyftem only be affected, and the vafcular pia mater completely pervious, topical irritation and tonic ftimuli would be ample means for exciting the abforbents to remove morbid accumulation. But if inflammation has once attacked the duplicatures of that membrane, or the encephalon itfelf, we can only depend upon local bleedings, brifk purges, mercurial frictions, naufeating dofes of antimony, &c. It is a difficult thing to afcertain the exiftence of an increafed fecretion at an early period of Hydrocephalus; and it is probable that practitioners fometimes miftake this ferious diforder for a trifling affection of the head. In order to guard againft an error of this kind as much as poffible, my firft object, whenever I am called to a child with the leaft apparent ailment of the brain, is to enquire the length of time it has been indifpofed; how it became fo? if there has been any imperfections in vifion? and whether fymptoms of fcrophula have ever been detected?

Had the illnefs been but of fhort duration, the pupils irritable, and the conftitution free from ftruma, I fhould conclude that no effufion of confequence had occurred, and my treatment would be regulated by the degree of fever, the particular nature of the complaint, and the feverity of pain. But if, on the contrary, my patient's health had been gradually declining with the appetite, habit relaxed, and the lymphatics affuming any peculiar ftate, I fhould ftrongly fufpect, provided there was indication of difeafe in the head, that Hydrocephalus was infidioufly gaining ground, and would, in a fhort time, produce extenfive mifchief.

It would be beft, in this cafe, I believe, to prefcribe blifters to the vertex, flight mercurial frictions, preparations of fteel, fea fea air and bathing, moderate exercife, &c. by which plan I fhould promote abforption, and fubdue the morbid action of the conftitution, in proportion as I gave ftrength, and rendered the circulation free. I have known a great many children perfectly reftored to health by purfuing this method; but I acknowledge, if it has been neglected in the beginning, and the fyftem has acquired a certain irritability, or local inflammation fupervened, it is not only then infufficient, but in my humble opinion rather injudicious. I wifh to remark, that I have feen *purgatives* at all times ferviceable, and particularly thofe of a powerful kind. The pulv. e fcam. cum calom. or other preparations of calomel, fucceed beft. Children always fuftain inteftinal evacuations exceedingly well; and as coftivenefs is one of the troublefome attendants upon this diforder, aperient medicines fhould be given pretty freely. A mercurial courfe is alfo commenced with much greater advantage, if abforption be previoufly excited by either local or general means.

*Tower, July* 7, 1802.

